# Probing the magnetic superexchange couplings between terminal Cu^II^ ions in heterotrinuclear bis(oxamidato) type complexes

**DOI:** 10.3762/bjnano.8.82

**Published:** 2017-04-06

**Authors:** Mohammad A Abdulmalic, Saddam Weheabby, Francois E Meva, Azar Aliabadi, Vladislav Kataev, Bernd Büchner, Frederik Schleife, Berthold Kersting, Tobias Rüffer

**Affiliations:** 1 Department of Inorganic Chemistry, Faculty of Natural Sciences, Chemnitz University of Technology, Strasse der Nationen 62, D-09111 Chemnitz, Germany; 2 Department of Pharmaceutical Sciences, Faculty of Medicine and Pharmaceutical Sciences, University of Douala, BP 2701, Cameroon; 3 Leibniz Institute for Solid State and Materials Research IFW Dresden, D-01171 Dresden, Germany; 4 Institut für Nanospektroskopie (EM-ISPEK), Helmholtz-Zentrum Berlin für Materialien und Energie, Kekuléstr. 5, D-12489 Berlin, Germany; 5 Institute for Solid State Physics, Technical University Dresden, D-01062 Dresden, Germany; 6 Department of Inorganic Chemistry, Faculty of Chemistry and Mineralogy, University of Leipzig, Johannisallee 29, D-04103 Leipzig, Germany

**Keywords:** bis(oxamidato), crystallographic characterization, diamagnetic, heteronuclear complexes, magnetic superexchange coupling, molecular structure

## Abstract

The reaction of one equivalent of [*n*-Bu_4_N]_2_[Ni(opboR_2_)] with two equivalents of [Cu(pmdta)(X)_2_] afforded the heterotrinuclear Cu^II^Ni^II^Cu^II^ containing bis(oxamidato) type complexes [Cu_2_Ni(opboR_2_)(pmdta)_2_]X_2_ (R = Me, X = NO_3_^–^ (**1**); R = Et, X = ClO_4_^–^ (**2**); R = *n-*Pr, X = NO_3_^–^ (**3**); opboR_2_ = *o*-phenylenebis(NR-substituted oxamidato); pmdta = *N*,*N*,*N*’,*N*”,*N*”-pentamethyldiethylenetriamine). The identities of the heterotrinuclear complexes **1**–**3** were established by IR spectroscopy, elemental analysis and single-crystal X-ray diffraction studies, which revealed the cationic complex fragments [Cu_2_Ni(opboR_2_)(pmdta)_2_]^2+^ as not involved in any further intermolecular interactions. As a consequence thereof, the complexes **1**–**3** possess terminal paramagnetic [Cu(pmdta)]^2+^ fragments separated by [Ni^II^(opboR_2_)]^2–^ bridging units representing diamagnetic *S*_Ni_ = 0 states. The magnetic field dependence of the magnetization *M*(*H*) of **1**–**3** at *T* = 1.8 K has been determined and is shown to be highly reproducible with the Brillouin function for an ideal paramagnetic spin = ^1^/_2_ system, verifying experimentally that no magnetic superexchange couplings exists between the terminal paramagnetic [Cu(pmdta)]^2+^ fragments. Susceptibility measurements versus temperature of **1**–**3** between 1.8–300 K were performed to reinforce the statement of the absence of magnetic superexchange couplings in these three heterotrinuclear complexes.

## Introduction

**Figure 1:**
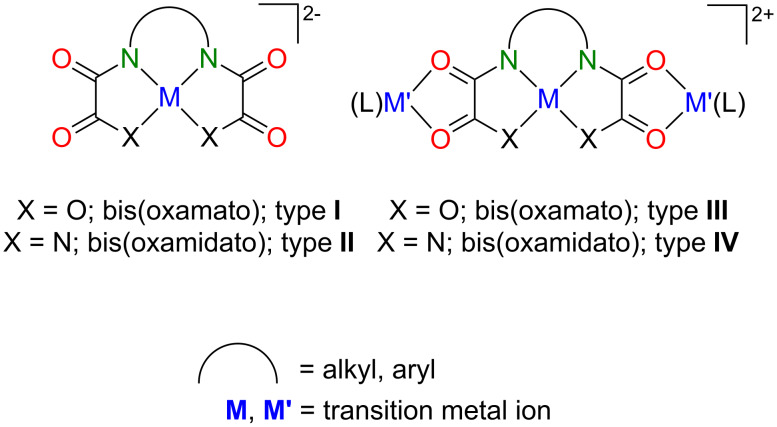
Chemical structures of type **I**–**IV** complexes.

Significant synthetic efforts have been directed to the synthesis of polynuclear species in which the metal ions are bridged by oxamato, oxamido, oxalato or dithiooxalato ligand [1–4]. In this context, the so-called bis(oxamato) type transition metal complexes as mononuclear species (Figure 1, type **I**) have received very special attention, as they allow the synthesis of multidimensional *n*D (*n* = 0–3) products, of which the magnetic properties were of specific interest [5]. Bis(oxamidato) type complexes (Figure 1, type **II**) have, on the other hand, received much less attention [6–9], although the flexidentate properties of these as well as type **I** complexes allows the convenient synthesis of the trinuclear type **III** and **IV** complexes, cf. Figure 1 [5,10–11].

The magnetic characterization of type **III** complexes has already significantly contributed to a better understanding of the origin of magnetic exchange interactions in polynuclear complexes [5,12]. One could expect that due to the lower electronegativity of the nitrogen atoms of type **III** (compared to the oxygen atoms of type **IV** complexes), the magnetic exchange couplings should increase [1]. These are studies to which we have already contributed [13–18].

**Figure 2:**
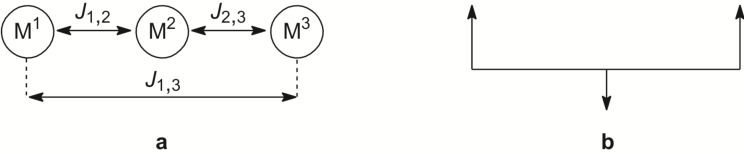
Expected *J* couplings between the central and terminal paramagnetic metal ions in type **III**/**IV** complexes (a). Approach of a small local spin located between two large local spins (b).

Basically, one can expect different magnetic exchange pathways between the paramagnetic metal ions of type **III** and **IV** complexes as depicted in Figure 2a and consequently these complexes might possess three different pathways in case that they are composed of three nonequivalent metal ions. To some extent, that has been already shown for heterotrinuclear Mn^II^Cu^II^Mn^II^ (*S* = ^9^/_2_) and Ni^II^Cu^II^Ni^II^ (*S* = ^3^/_2_) type **III** complexes [19–21]. Thus, by locating a small local between two large spins (Figure 2b), complexes with high-spin ground states can been obtained.

If we follow this idea further we could replace the middle local spin, cf. Figure 2b, by a diamagnetic fragment. This would allow unambiguous verification of whether type **III**/**IV** complexes might have *J*_1,3_ magnetic couplings (Figure 2a) or not. There is already a first study of Sanada et al. [11], who reported for the heterotrinuclear Gd^III^Ni^II^Gd^III^ type **IV** complex (*S* = ^7^/_2_) a very small *J*_1,3_ coupling of −0.002 cm^–1^. However, this small coupling might be attributed to the shielding effect of the outer-shell electrons on the 4f electron of the Gd^III^ ions [11]. On the other hand, for homotrinuclear Cu^II^Cu^II^Cu^II^ type **III** complexes, *J*_1,3_ couplings were either assumed to be zero or negligible [14–16,22]. One can thus conclude that *J*_1,3_ couplings are very small.

In our earlier work, we previously reported on the magnetic characterization of homotrinuclear Cu^II^Cu^II^Cu^II^ type **IV** complexes [15]. We noticed, unexpectedly, that the central Cu^II^ ions of these complexes were not coordinated by any counter ions or solvents. It is this finding which gave birth to the idea to report here on the synthesis of heterotrinuclear Cu^II^Ni^II^Cu^II^ type **IV** complexes. Their central [Ni^II^(opboR_2_)]^2–^ fragments were anticipated to be free of any further co-ligands. That would make these central fragments purely diamagnetic and thus these heterotrinuclear Cu^II^Ni^II^Cu^II^ type **IV** complexes, possessing terminal paramagnetic Cu^II^ ions, appear as ideal candidates to study the magnitude of the *J*_1,3_ coupling of type **III**/**IV** complexes.

## Results and Discussion

### Synthesis

**Scheme 1:**
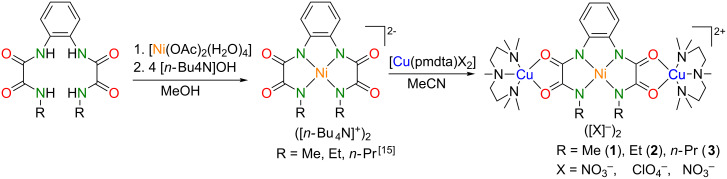
Synthesis of the heterotrinuclear Cu^II^Ni^II^Cu^II^ type **IV** complexes **1**–**3**.

The synthesis of the heterotrinuclear Cu^II^Ni^II^Cu^II^ complexes **1**–**3** out of literature-known precursors is shown in Scheme 1.

Under anaerobic working conditions one equivalent of the non-hygroscopic [*n-*Bu_4_N]^+^ salts of mononuclear [Ni^II^(opboR_2_)]^2–^ complexes were treated with two equivalents of [Cu(pmdta)(X)_2_] (X = NO_3_^–^ for **1** and **3**, X = ClO_4_^–^ for **2**) in MeCN solutions of to give [NiCu_2_(opboR)(pmdta)_2_](X)_2_ (**1**–**3**, cf. Scheme 1) in yields exceeding 60%. The reaction side products [*n*-Bu_4_N][NO_3_] and [*n-*Bu_4_N][ClO_4_], respectively, could be smoothly separated as they are soluble in 4:1 THF/Et_2_O mixtures, while the desired complexes **1**–**3** are insoluble in such mixtures. The isolated powders of **1**–**3** had to be stored under inert gas atmosphere, as they are hygroscopic. Single crystals of **1**–**3** could be obtained as described next by crystallisation experiments performed under inert atmosphere.

### Single crystal X-ray diffraction studies

Slow diffusion of Et_2_O vapour into CH_2_Cl_2_ solutions of **1** and **3** and into a MeCN solution of **2** afforded single crystals suitable for crystallographic studies of the compositions [{NiCu_2_(opboMe_2_)(pmdta)_2_}_2_][NO_3_]_4_·3.75CH_2_Cl_2_ (**1’**), [NiCu_2_(opboMe_2_)(pmdta)_2_][ClO_4_]_2_·2MeCN (**2’**) and [NiCu_2_(opboMe_2_)(pmdta)_2_][NO_3_]_2_·2CH_2_Cl_2_ (**3’**). In case of **1’**, the asymmetric unit comprises two crystallographically independent complexes of **1**. Their dicationic complex fragments [Cu_2_Ni(opboMe_2_)(pmdta)_2_]^2+^ are denoted in the following as **1A** (comprising Ni1) and **1B** (comprising Ni2). The related bond lengths and angles of **1A**/**1B** show differences of up to 1.5% and ca. 2%, respectively, whereby only bond lengths and angles of **1A** will be discussed, although Table 1 and Table 2 displays them for both **1A** and **1B**. In analogy, the cationic complex fragments [NiCu_2_(opboMe_2_)(pmdta)_2_]^2+^ of **2’** and [NiCu_2_(opboMe_2_)(pmdta)_2_]^2+^ of **3’** are denoted in the following as **2A** and **3A**. It should be highlighted and emphasized that in the crystal structures of **1’**–**3’** no unusual short intermolecular interactions were observed and that the complex fragments **1A**–**3A** are indeed discrete.

**Table 1:** Selected bond lengths (Å) and angles (°) of the [Ni(opboR_2_)]^2–^ fragments of **1A**/**1B** (R = Me), **2A** (R = Et) and **3A** (R = *n*-Pr).

	**1A**/**1B**	**2A**	**3A**

Bond lengths			

N1–Ni1	1.865(6)/1.862(6)	1.869(6)	1.847(5)
N2–Ni1	1.915(6)/1.912(7)	1.922(6)	1.904(6)
N3–Ni1(N1A–Ni1)^a^	1.866(6)/1.867(6)	1.860(6)	1.847(5)
N4–Ni1(N2A–Ni1)^a^	1.923(6)/1.922(7)	1.923(6)	1.904(6)
C1–O1	1.260(8)/1.238(9)	1.259(10)	1.264(8)
C2–O2	1.280(8)/1.300(9)	1.261(9)	1.289(8)
C3–O3(C1A–O1A)^a^	1.255(8)/1.248(9)	1.237(9)	1.264(8)
C4–O4 (C2A–O2A)^a^	1.279(8)/1.280(9)	1.278(9)	1.289(8)
C1–N1	1.316(9)/1.325(10)	1.315(10)	1.317(8)
C2–N2	1.280(9)/1.288(10)	1.333(10)	1.326(9)
C3–N3(C1A–N1A)^a^	1.308(9)/1.303(10)	1.331(10)	1.317(8)
C4–N4(C2A–N2A)^a^	1.304(9)/1.281(11)	1.298(10)	1.326(9)
C1–C2	1.535(9)/1.522(10)	1.492(11)	1.487(11)
C3–C4(C1A–C2A)^a^	1.514(9)/1.533(10)	1.515(11)	1.487(11)

Bond angles			

N1–Ni1–N3(N1–Ni1–N1A)^a^	83.7(2)/83.7(3)	83.7(3)	83.5(3)
N2–Ni1–N4(N2–Ni1–N2A)^a^	107.1(2)/107.5(3)	107.5(3)	107.0(4)
N1–Ni1–N2	84.6(2)/84.4(3)	84.4(3)	85.0(2)
N3–Ni1–N4(N1A–Ni1–N2A)^a^	84.7(2)/84.3(3)	84.5(3)	85.0(2)
N1–Ni1–N4(N1–Ni1–N2A)^a^	167.8(2)/167.6(3)	168.0(3)	166.9(2)
N2–Ni1–N3(N2–Ni1–N1A)^a^	168.2(2)/168.0(3)	167.9(3)	166.9(2)
N1–C1–O1	129.7(6)/129.9(7)	128.7(8)	128.2(7)
N2–C2–O2	127.1(6)/126.7(7)	125.0(7)	126.3(7)
N3–C3–O3(N1A–C1A–O1A)^a^	129.6(6)/129.5(7)	128.7(7)	128.2(7)
N4–C4–O4(N2A–C2A–O2A)^a^	126.1(6)/127.8(7)	126.9(7)	126.3(7)

^a^Data in brackets refer to respective bond lengths and angles of **10A**. Symmetry operation used to generate equivalent atoms ‘A’ for **10A**: –x, y, –z + ^3^/_2_.

**Table 2:** Selected bond lengths (Å), angles (°) and τ parameters of the terminal [Cu(pmdta)]^2+^ fragments of **1A**/**1B** (R = Me), **2A** (R = Et) and **3A** (R = *n-*Pr).

	**1A**/**1B**	**2A**	**3A**

Bond lengths			

Cu1–O1	2.243(5)/2.218(5)	2.210(5)	2.192(5)
Cu1–O2	1.957(5)/1.953(5)	1.998(6)	1.982(5)
Cu1–N5	2.047(6)/2.061(6)	2.070(8)	2.077(6)
Cu1–N6	2.028(7)/1.997(7)	2.015(7)	2.010(6)
Cu1–N7	2.072(6)/2.090(6)	2.035(7)	2.029(6)
Cu2–O3	2.198(5)/2.203(5)	2.198(5)	–^a^
Cu2–O4	1.962(5)/1.957(6)	1.994(5)	–^a^
Cu2–N8	2.042(6)/2.044(7)	2.056(6)	–^a^
Cu2–N9	2.010(6)/2.007(10)	2.014(6)	–^a^
Cu2–N10	2.091(6)/2.082(9)	2.072(7)	–^a^

Bond angles			

O1–Cu1–O2	81.78(17)/82.2(2)	81.1(2)	81.80(19)
O1–Cu1–N5	99.1(2)/98.7(3)	99.5(3)	99.5(2)
O1–Cu1–N6	105.9(2)/104.1(2)	104.3(3)	101.6(2)
O1–Cu1–N7	103.7(2)/105.6(2)	106.4(3)	107.6(2)
O2–Cu1–N5	94.0(2)/95.0(3)	92.8(3)	92.9(2)
O2–Cu1–N6	172.0(2)/173.1(2)	174.6(3)	176.6(3)
O2–Cu1–N7	90.4(2)/89.7(3)	91.8(3)	92.6(2)
N5–Cu1–N6	87.0(3)/86.9(3)	86.3(3)	86.3(3)
N5–Cu1–N7	157.2(3)/155.6(3)	154.1(3)	152.8(3)
N6–Cu1–N7	85.6(3)/86.0(3)	86.7(3)	86.7(3)
O3–Cu2–O4	81.75(18)/81.9(2)	81.4(2)	–^a^
O3–Cu2–N8	101.0(2)/102.0(3)	104.2(2)	–^a^
O3–Cu2–N9	104.4(2)/105.3(3)	102.8(2)	–^a^
O3–Cu2–N10	101.8(2)/100.5(3)	100.2(2)	–^a^
O4–Cu2–N8	92.4(2)/91.3(3)	92.5(2)	–^a^
O4–Cu2–N9	173.8(2)/172.8(3)	175.8(3)	–^a^
O4–Cu2–N10	92.4(2)/90.3(4)	93.0(2)	–^a^
N8–Cu2–N9	86.5(2)/86.5(4)	86.2(3)	–^a^
N8–Cu2–N10	157.1(3)/157.4(3)	155.6(3)	–^a^
N9–Cu2–N10 τ parameter Cu1	86.3(3)/89.1(5) 0.247/0.292	86.6(3) 0.342	–^a^ 0.397
Cu2	0.278/0256	0.337	–^a^

^a^Data of this [Cu(pmdta)]^2+^ fragment corresponds to those of the [Cu(pmdta)]^2+^ fragment comprising the atom Cu1, due to the crystallographically imposed *C*_2_ symmetry of **3A**.

**Figure 3:**
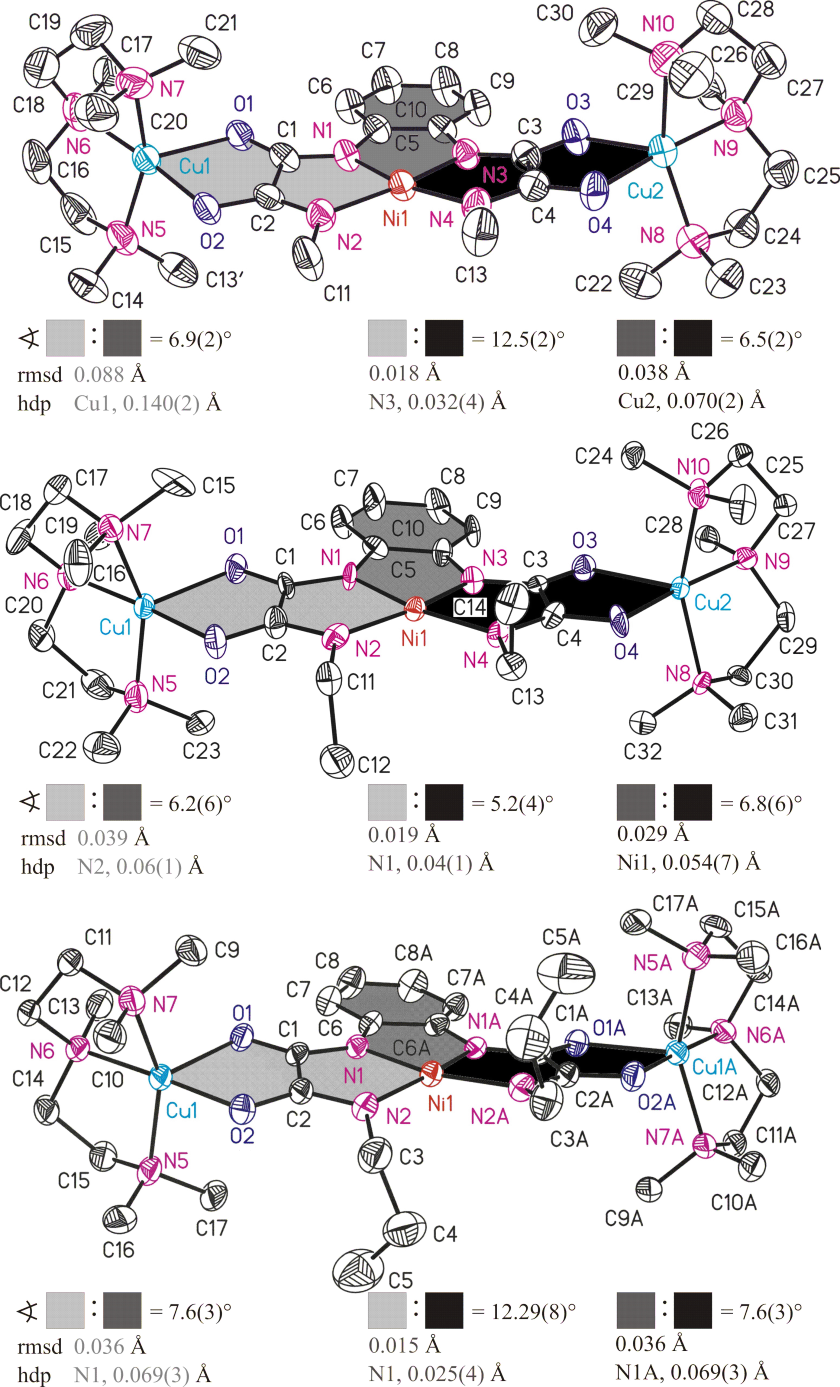
ORTEP diagrams (50% ellipsoid probability) of the molecular structures of **1A** (top), **2A** (middle) and **3A** (down), respectively. All hydrogen atoms are omitted for clarity. The sign 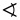 refers to the interplanar angle, rmsd to the root-mean-square deviation from planarity and hdp to the highest deviation from planarity of calculated mean planes of atoms adjoining differently coloured areas. Symmetry code ‘A’ for 10A: –x, y, –z + ^3^/_2_. The rmsd/hdp of atoms adjoining light gray and black coloured areas amounts as follows: 8A, 0.118 Å/Cu1 with 0.410 Å. 9A, 0.064 Å/O2 with 0.135 Å. 10A, 0.107 Å/O2 with 0.207 Å.

The molecular structures of **1A**–**3A** are similar to each other and thus structural features of all three complex fragments will be discussed together. A collective plot of the molecular structures of **1A**–**3A** in an analogous perspective view is shown in Figure 3. Selected bond lengths and angles of the [Ni(opboR_2_)]^2–^ and of the [Cu(pmdta)]^2+^ complex fragments of **1A**–**3A** are given in Table 1 and Table 2, respectively. Crystal and structural refinement data are summarized in Table 3.

**Table 3:** Crystal and structural refinement data of **1’**, **2’**and **3’**.

	**1’**	**2’**	**3’**

Empirical formula	C_255_H_478_Cl_30_Cu_16_N_96_Ni_8_O_80_	C_36_H_66_Cl_2_Cu_2_N_12_NiO_12_	C_36_H_68_Cl_4_Cu_2_N_12_NiO_10_
Formula weight (g·mol^–1^)	8719.15	1115.69	1156.61
Crystal system	orthorhombic	triclinic	orthorhombic
Space group	*Pbca*	*P−1*	*Pbcn*
*a* (Å)	35.6630(14)	8.2749(3)	29.485(2)
*b* (Å)	14.0366(7)	10.9892(3)	11.2595(13)
*c* (Å)	37.5448(16)	30.1863(10)	16.0310(13)
α (°)	90.0	83.352(3)	90.0
β (°)	90.0	82.706(3)	90.0
γ (°)	90.0	70.178(3)	90.0
*V* (Å^−3^)	18810.3(14)	2553.65(15)	5322.1(8)
Measurement temperature (K)	110	115	110
Radiation source	Cu Kα	Cu Kα	Cu Kα
Wavelength (Å)	1.54184	1.54184	1.54184
*Z*	2	2	4
Density (calculated) (Mg·m^–3^)	1.539	1.451	1.443
Absorption coefficient (mm^–1^)	4.008	2.912	3.687
*F*(000)	9036	1164	2408
Reflections collected	53695	10264	10911
Reflections unique /*R*_int_^a^	15422, 0.0475	10264, 0.0412	4212, 0.0462
Limiting indices	–23 ≤ *h* ≤ 41,	–9 ≤ *h* ≤ 9,	–32≤ *h* ≤ 34,
	–16 ≤ *k* ≤ 11,	–12 ≤ *k* ≤ 12,	–12 ≤ *k* ≤ 12,
	–43 ≤ *l* ≤ 42	–34 ≤ *l* ≤ 31	–18 ≤ *l* ≤ 15
θ range for data collection (°)	3.417 to 62.981	4.290 to 62.706	4.203 to 62.744
Data/restraints/parameters	15422/1164/1126	10264/662/645	4212/289/292
Goodness-of-fit on *F*^2 b^	0.938	1.101	0.830
Final *R* indices [*I* > 2σ(*I*)]^c^	*R*_1_ = 0.0816, *wR*_2_ = 0.2279	*R*_1_ = 0.0777, *wR*_2_ = 0.2073	*R*_1_ = 0.0810, *wR*_2_ = 0.2120
*R* indices (all data)^c^	*R*_1_ = 0.1234, *wR*_2_ = 0.2460	*R*_1_ = 0.0816, *wR*_2_ = 0.2097	*R*_1_ = 0.1364, *wR*_2_ = 0.2337
Largest diff. peak/hole (e·Å^–3^)	1.988/−1.311	1.132/−0.549	0.925/−0.868

^a^*R*_int_ = Σ│*F*_o_^2^–*F*_o_^2^(mean)│/Σ*F*_o_^2^, where *F*_o_^2^(mean) is the average intensity of symmetry equivalent diffractions. ^b^*S* = [∑*w*(*F*_o_^2^ – *F*_c_^2^)^2^]/(*n – p*)^1/2^, where *n* = number of reflections, *p* = number of parameters. ^c^*R* = [∑(||*F*_o_| – |*F*_c_|)/∑|*F*_o_|); *wR* = [∑(*w*(*F*_o_^2^ – *F*_c_^2^)^2^)/∑(*wF*_o_^4^)]^1/2^.

The Ni^II^ ions of **1A**–**3A** are coordinated by four deprotonated amide N donor atoms to form a planar-quadratic NiN_4_ coordination environment. Two of them belong to the *N*,*N*’-*o*-phenylene bridges of **1A**–**3A** (**1A/2A**: N1 and N3. **3A**: N1 and N1A) and are referred to in the following as N_aryl_ donor atoms. The other two belong to the alkyl-substituted amide functions of **1A**–**3A** (**1A**/**2A**: N2 and N4. **3A**: N2 and N2A) and are further referred to as N_alkyl_ donor atoms. The planarity of the NiN_4_ units is revealed, for example, by calculations of mean planes of its atoms and gives the following root-mean-square deviations from planarity (rmsd) together with values for the atom with the highest deviation from planarity (hdp) as follows: **1A**/**2A**/**3A** (rmsd, hdp) = 0.035 Å, N1 with 0.046(3) Å /0.030 Å, N1 with 0.035(8) Å/0.082 Å, N1 with 0.100(4) Å, respectively. Moreover, the sum of bond angles of the NiN_4_ units amounts to 360.1(4)° (**1A**), 360.1(6)° (**2A**) and 360.5(5)° (**3A**). For the mononuclear Ni^II^-containing bis(oxamato) complex [*n*-Bu_4_N]_2_[Ni(opba)] (**11**) [23] and the related bis(oxamidato) type complex [Ph_4_P]_2_[Ni(opboMe_2_)] (**12**) [9] the following observation has been made: Three of bond angles of the central NiN_2_O_2_/NiN_4_ coordination units are small (**11**: 85.79(8)–86.18(5)°; **12**: 82.7(3)–84.7(3)°), while the fourth one is significantly larger (**11**: 101.97(7)°; **12**: 108.8(3)°). Thereby, the latter bond angle is the one created of the two carboxylate oxygen atoms of **11** or the two N_alkyl_ donor atoms of **12**. This feature is due to the presence of 5-5-5 fused chelate rings around the Ni^II^ ion [17,24]. In case of **1A**–**3A** this feature is observed as well, cf. Table 1.

The Ni–N bond lengths of the NiN_4_ units of **1A**–**3A** fall into two categories: The Ni–N_aryl_ bond lengths are significantly shorter compared to the Ni–N_alkyl_ ones [25]. For example, the Ni–N_aryl_ bond lengths of **1A** (Ni1–N1 and Ni1–N3, 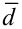
= 1.864(8) Å) are substantially shorter compared to the Ni–N_alkyl_ bond lengths (Ni1–N2 and Ni1–N4, 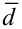 = 1.912(8) Å). This fact is in principal in agreement with the observations made for **12** [9] and could be explained in analogy to statements made for mononuclear Cu^II^-containing bis(oxamato) complexes by the greater basicity of the N_aryl_ vs the N_alkyl_ donor atoms [24].

In the following the geometries of the terminal [Cu(pmdta)]^2+^ fragments will be briefly described. It should be emphasized that the findings described in the following have been made analogously for our previously reported homotrinuclear Cu^II^Cu^II^Cu^II^ complexes as described in [15]. Thus, the terminal Cu^II^ ions of **1A**–**3A** are each coordinated by two O donor atoms of the oxamidato groups as well as three N donor atoms of the pmdta ligands to form CuN_3_O_2_ coordination units closer to the ideal square-pyramidal compared to the ideal trigonal-bipyramidal coordination geometry with respect to their τ parameters [26], cf. Table 2. One feature, commonly observed for all CuN_3_O_2_ units, deserves specific attention. The largest bond angle of all CuN_3_O_2_ units always involves the O donor atom of the 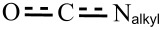 function and the middle N donor atom of the pmdta ligands, cf. Figure 1 and Table 2. A related observation was made recently for the asymmetric trinuclear complex [Cu_3_(opooMe)(pmdta)_2_](NO_3_)_2_ (**13**, opooMe = *o*-phenylene(*N’*-methyl oxamidato)(oxamato)) [13] and has been compared to observations made for bis(oxamato) type entities. As observed for the CuN_3_O_2_ units of **1A**–**3A**, even in the case of **13**, the largest O–Cu–N bond angle involves the O donor atom of the 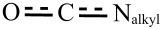 function for the oxamidato side, whereas in case of the oxamato side the largest bond angle involves the O donor atom of the 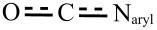 function. Consequences of this observation to magnetic exchange couplings have been discussed [13]. Thus, it seems that for polynuclear complexes comprising one or two oxamidato groups, cf. [13] and [15], this specific feature of the terminal CuN_3_O_2_ units is of broader validity.

It is recalled that the Ni^II^ ions of **1A**–**3A** are not coordinated further by any counter anions and/or solvent molecules. In contrast, in Cu^II^Cu^II^Cu^II^ type **III** complexes (Figure 1) the central Cu^II^ ions are commonly further coordinated, even by BF_4_^–^ ions [14]. Hence, the Ni^II^ ions of **1A**–**3A** indeed represent diamagnetic *S*_Ni_ = 0 states. Specifically, this property makes them excellently suited candidates to experimentally verify whether long-range magnetic superexchange interactions along two consecutively aligned oxamidato and even oxamato bridges are possible.

### Magnetic properties

**Figure 4:**
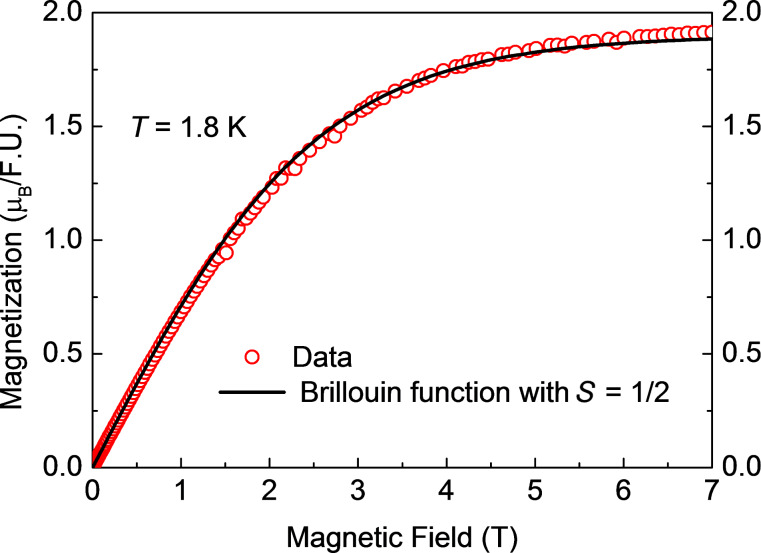
Magnetization versus magnetic field *M*(*H*) of **1** at *T* = 1.8 K (symbols) together with the fit of *M*(*H*) to the Brillouin function with *S* = ^1^/_2_ according to Equation 1 (solid line).

**Figure 5:**
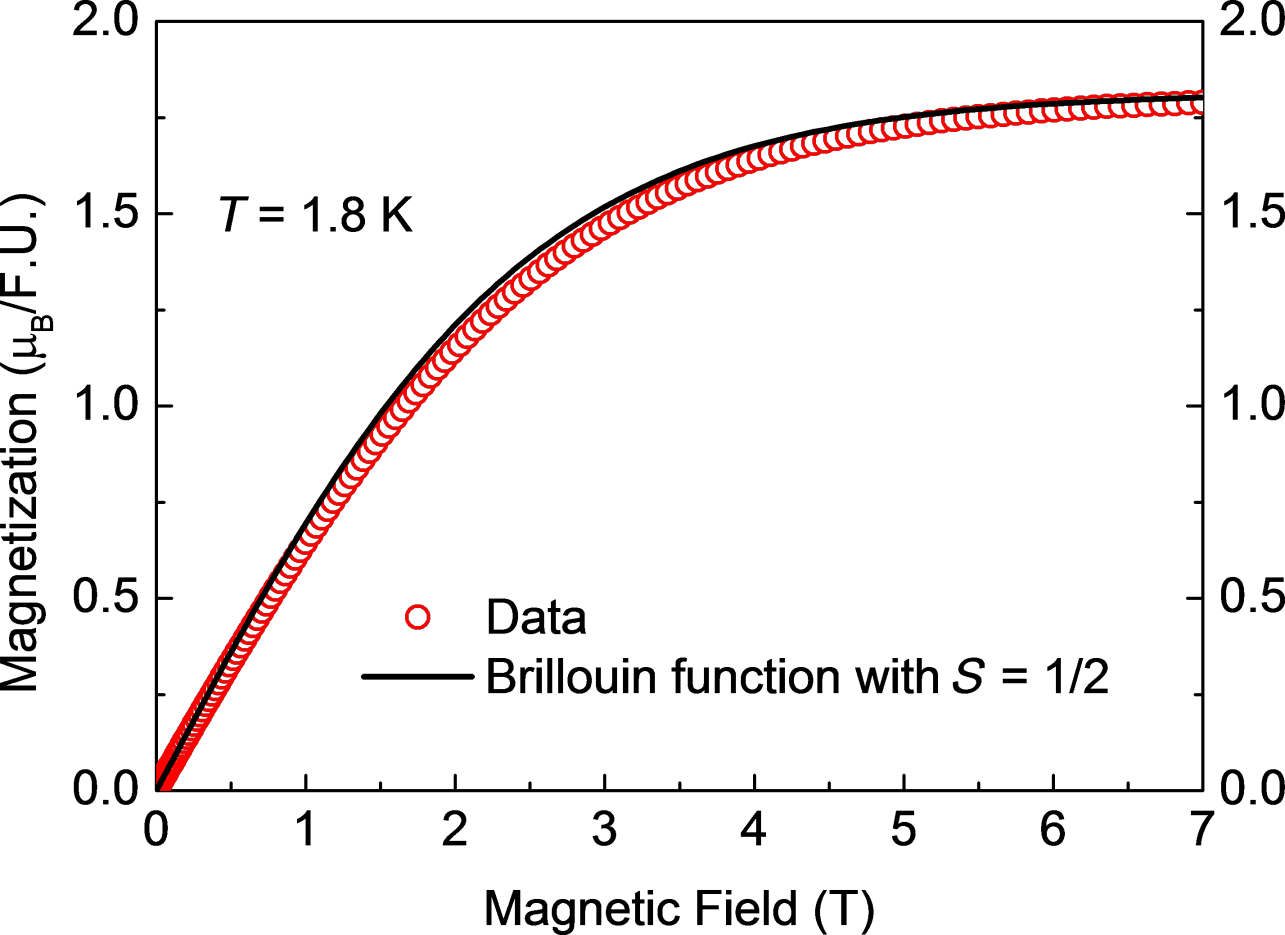
Magnetization versus magnetic field *M*(*H*) of **2** at *T* = 1.8 K (symbols) together with the fit of *M*(*H*) to the Brillouin function with *S* = ^1^/_2_ according to Equation 1 (solid line).

**Figure 6:**
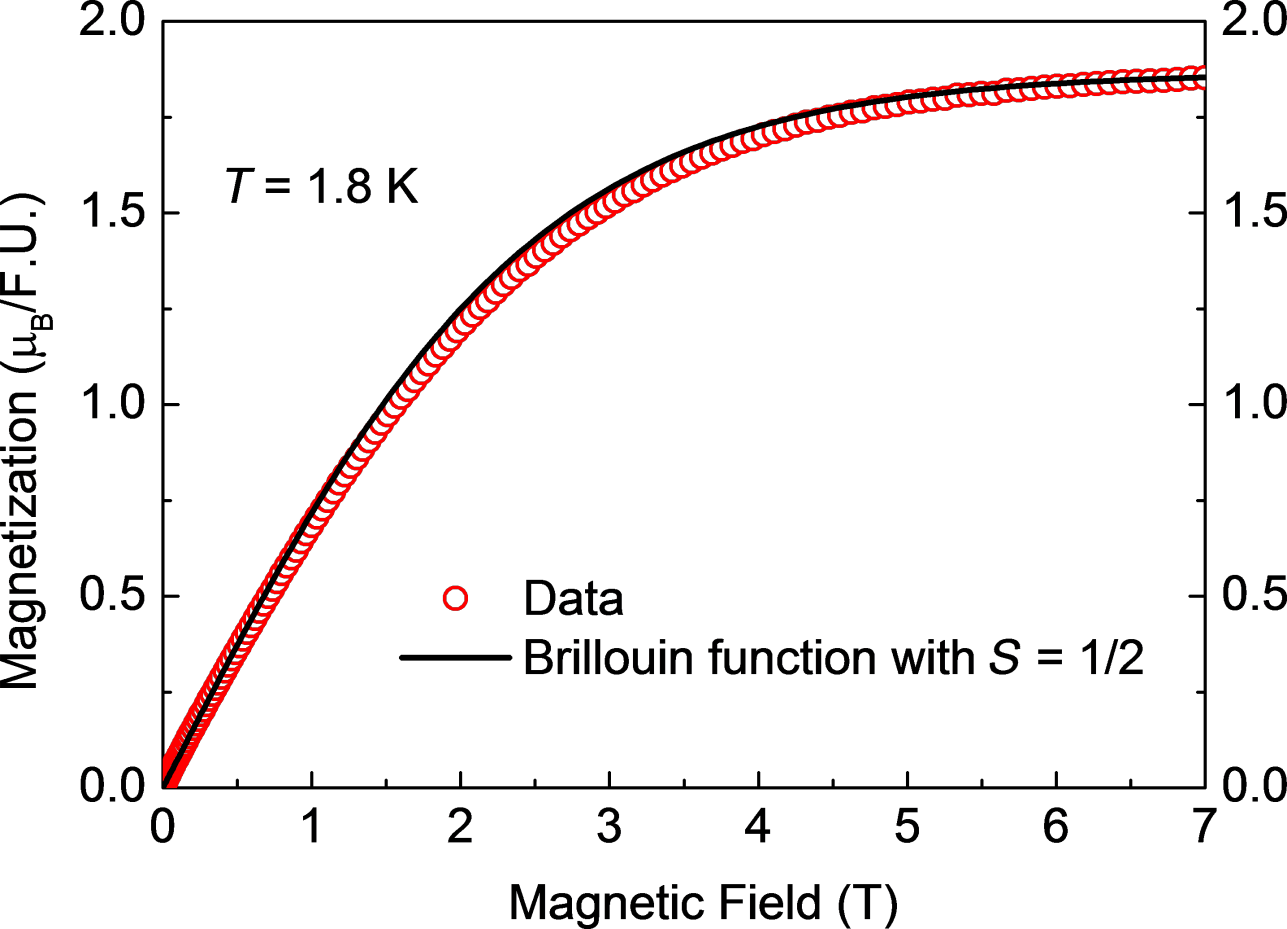
Magnetization versus magnetic field *M*(*H*) of **3** at *T* = 1.8 K (symbols) together with the fit of *M*(*H*) to the Brillouin function with *S* = ^1^/_2_ according to Equation 1 (solid line).

The results of the measurements of the magnetic field dependence of the magnetization *M*(*H*) for samples **1**, **2** and **3** at *T* = 1.8 K are shown in Figure 4, Figure 5 and Figure 6. All curves can be very well fitted with the Brillouin function for spin *S* = ^1^/_2_ and the spectroscopic *g*-factor *g* = 2.1 determined from the electron spin resonance (ESR) spectra (not shown):

[1]
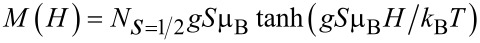


Here, *N_S_*_=1/2_ is the number of spins ^1^/_2_ in the molecule, µ_B_ is the Bohr magneton, and *k*_B_ is the Boltzmann constant. Considering that Equation 1 describes the behavior of an ideal paramagnet comprising non-interacting spins and that Equation 1 nicely reproduces the shape of the measured *M*(*H*) dependences, one can safely conclude that at *T* = 1.8 K and (within the experimental uncertainty) there is no magnetic interaction between the Cu^II^ spins of the terminal [Cu(pmdta)]^2+^ complex fragments in all three samples. At fields above 5 T, all *M*(*H*) curves saturate, cf. Figure 4–6. Under these experimental conditions one has *gS*µ_B_*H*/*k*_B_*T* >> 1 and Equation 1 thus reduces to *M*_sat_(*H*) = *N_S_*_=1/2_*gS*µ_B_ for the heterotrinuclear **1**–**3** with *N_S_*_=1/2_ = 2. Therefore, the expected saturation magnetization for *S* = ^1^/_2_ and *g* = 2.1 should amount to *M*_sat_(*H*) = 2.1µ_B_ per formula unit (f.u.). The experimentally observed values of *M*_sat_(*H*) are somewhat smaller, amounting to 1.91µ_B_, 1.79µ_B_, and 1.85µ_B_ for **1**, **2** and **3**, respectively. This implies that the effective number of non-interacting Cu^II^ spins per f.u. which contribute to the magnetization signal is smaller than *N_S_*_=1/2_ = 2 and amounts to 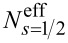 = 1.82, 1.7, and 1.76 for **1**, **2** and **3**, respectively. This discrepancy of the order of ≈10% in average could be attributed to remaining amounts of packing solvent molecules and thus errors in the determination of the molecular weight. It could be attributed furthermore to the hygroscopic nature of vacuum-dried single crystals of **1’**–**3’** and as the sample preparation was performed under aerobic conditions, cf. Experimental Section and Supporting Information File 1, giving thus errors in the determination of the molecular weight of the samples.

**Figure 7:**
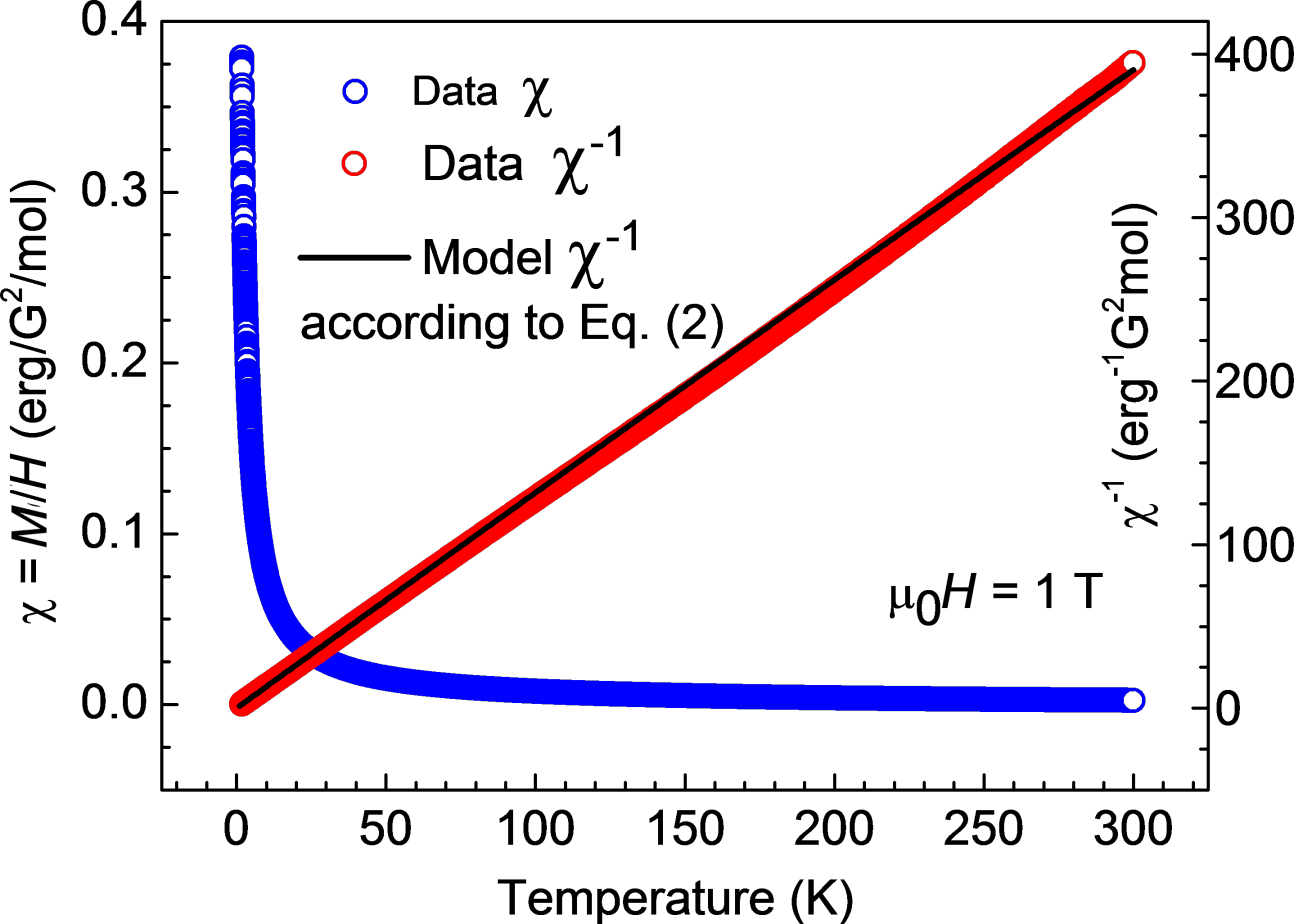
Temperature dependence of the magnetic susceptibility χ = *M*/*H* and of the corresponding inverse susceptibility χ^−1^ for **1** (symbols). The black line represents a model curve 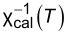 according to Equation 2 (see the text).

**Figure 8:**
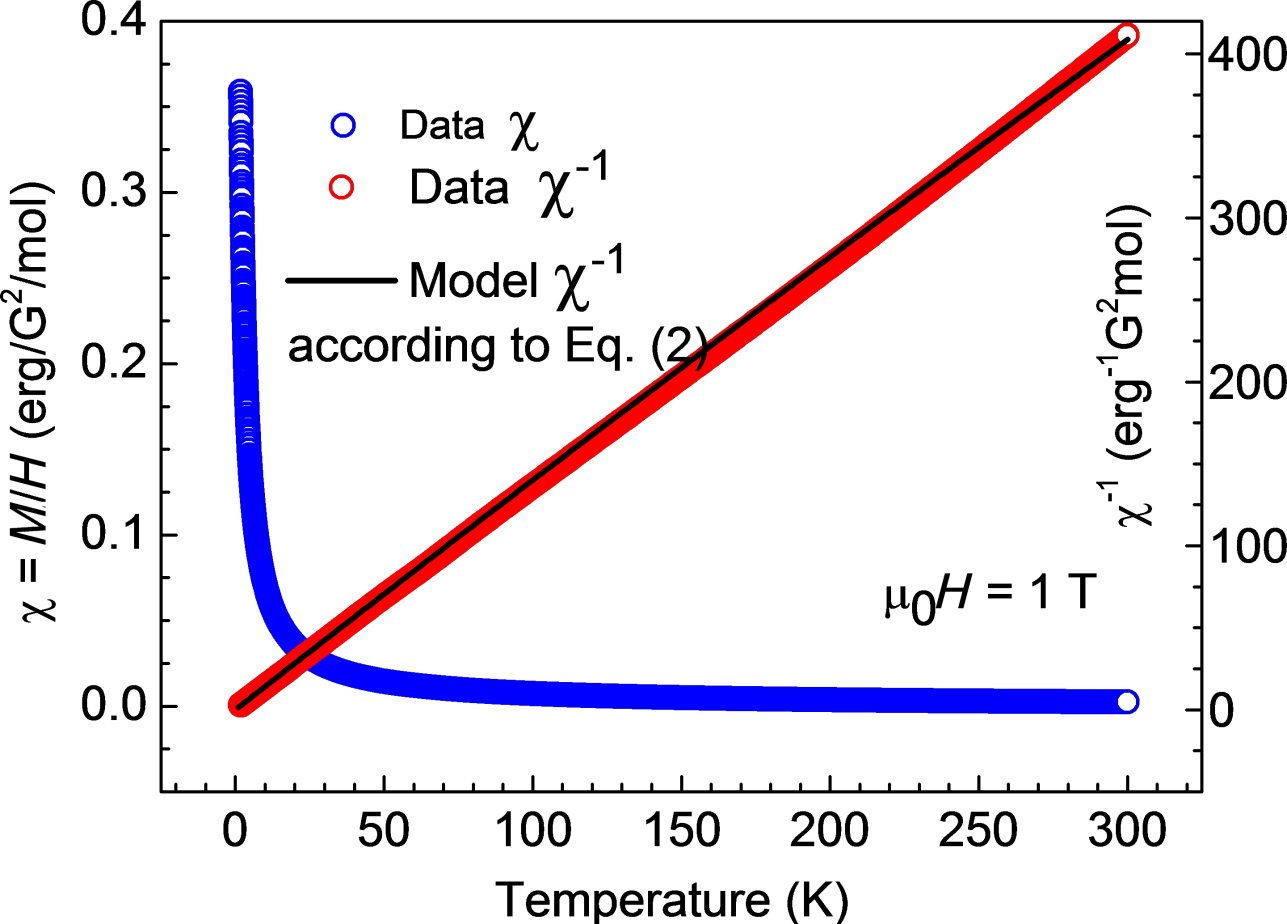
Temperature dependence of the magnetic susceptibility χ = *M*/*H* and of the corresponding inverse susceptibility χ^−1^ for **2** (symbols). The black line represents a model curve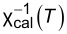 according to Equation 2 (see the text).

**Figure 9:**
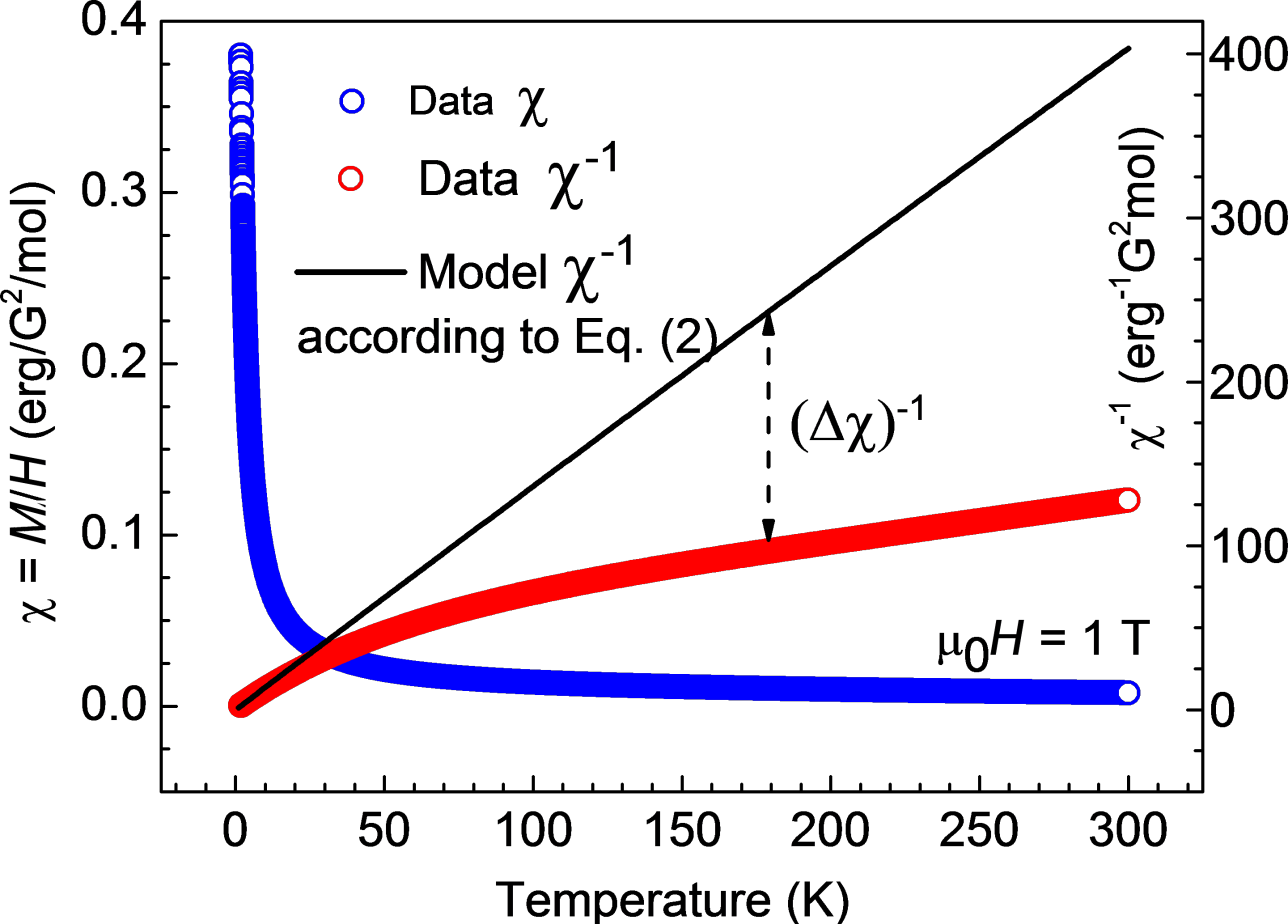
Temperature dependence of the magnetic susceptibility χ = *M*/*H* and of the corresponding inverse susceptibility χ^−1^ for **3**. The black line represents a model curve 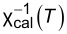 according to Equation 2 (see the text). The dashed arrow indicates the discrepancy between the model curve and the experimental dependence.

Further insights into the magnetism of the studied samples can be obtained from the analysis of the temperature dependence of the static magnetic susceptibility χ = *M*/*H*. The curves χ(*T*) and the corresponding inverse susceptibility χ^−1^(*T*) for **1**, **2** and **3** are presented in Figure 7–9. These dependences for **1** and **2** can be very well understood in terms of the Curie–Weiss law:

[2]
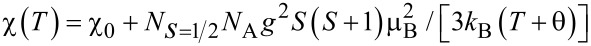


Here, χ_0_ is a temperature independent term comprising the van Vleck and diamagnetic susceptibilities, *N*_A_ is the Avogadro number, and θ is the Curie–Weiss temperature which is a measure of the magnetic interaction between the spins. Since the analysis of the *M*(*H*) curves reveal no interaction between Cu^II^ spins, θ can be assumed zero. With *S* = ^1^/_2_, *g* = 2.1 and the values of 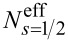 from the saturation magnetization *M*_sat_(*H*) one can calculate the dependence (Equation 2) versus 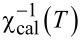 as plotted in black in Figure 7 and Figure 8. Obviously, the plots agree well with the experimental dependence χ^−1^(*T*) for **1** and **2**. Here, the values χ_0_ = 5·10^–5^ erg/G^2^/mol and 1·10^–4^ erg/G^2^/mol were chosen for samples **1** and **2**, respectively. From the above discussion one can therefore conclude that the self-consistent analysis of the *M*(*H*) and χ(*T*) dependences gives evidence for the absence of magnetic interaction between the terminal Cu^II^ ions in the heterotrinuclear Cu^II^Ni^II^Cu^II^ complexes **1** and **2**.

**Figure 10:**
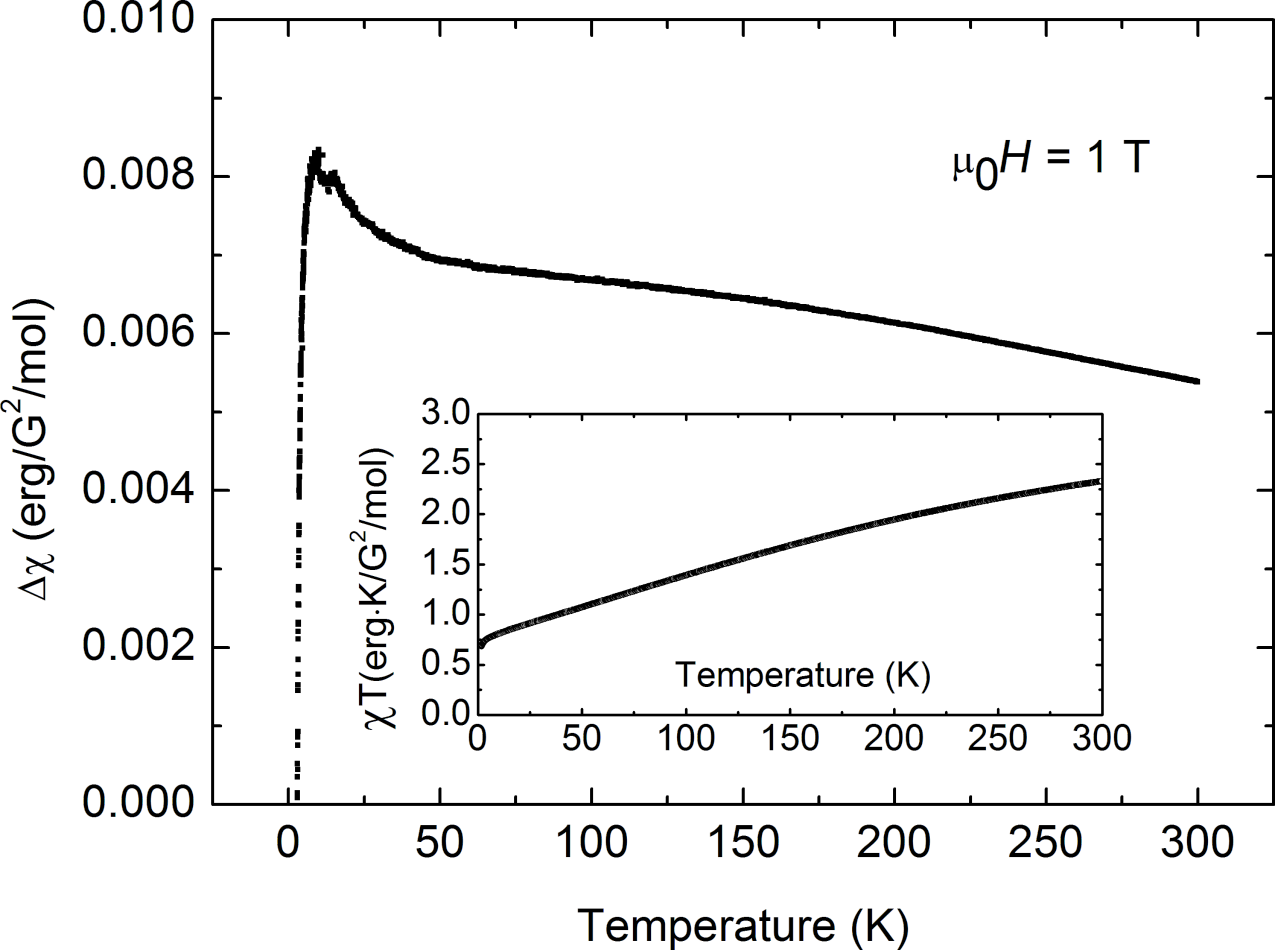
Main panel: Difference between the calculated and measured static susceptibility for **3**. Inset: Temperature dependence of the product χ*T* for **3** (see the text).

Unfortunately, no definite conclusion can be drawn for complex **3**. The similarly calculated curve 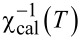 according to Equation 2 is shown by the black solid curve in Figure 9. It strongly deviates from the measured χ^−1^(*T*) dependence. Correspondingly, the product χ(*T*)*T* increases with temperature (Figure 10, inset). There is obviously an additional contribution to the static susceptibility, leading to lower values of the inverse susceptibility 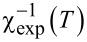 of the sample. This contribution is absent in the magnetization data at *T* = 1.8 K, suggesting that it may originate from some species in a concentration of the order of 10% with thermally activated magnetism. The difference Δχ = χ_exp_ − χ_cal_ is plotted in Figure 10, main panel, and might originate from paramagnetic impurities, cf. [16]. On the other hand, vacuum-dried powders of **3’** appeared as more hygroscopic compared to the ones of **1’** and **2’**, cf. above and Supporting Information File 1. As the sample preparation was performed under aerobic conditions, it is imaginable that air moisture had an impact on these measured as it is shown for the IR spectroscopically characterized **3**. Attempts to model this contribution with some specific models invoking possible exchange interactions between the two Cu centers (e.g., [13,15,27]) were not successful.

## Conclusion

The three heterotrinuclear bis(oxamidato) type complexes comprising [Cu_2_Ni(opboR_2_)]^2+^ fragments (R = Me (**1**), Et (**2**), *n-*Pr (**3**)) could be successfully synthesized and their identities have been unambiguously established by single-crystal X-ray diffraction studies. These studies revealed that all [Cu_2_Ni(opboR_2_)]^2+^ fragments are not involved in any intermolecular interactions and are thus discrete in the solid state. That made these three complexes especially well-suited to experimentally verify that there are no magnetic superexchange couplings between their terminal [Cu(pmdta)]^2+^ fragments. Thus, we can conclude that for trinuclear type **IV** as well as type **III** complexes incorporating exclusively 3d transition metal ions, no long-range magnetic couplings across two consecutively aligned oxamidato or oxamato bridges can occur.

## Experimental

### General methods and materials

All chemicals were purchased from commercial sources and used as received unless stated otherwise. All reactions were carried out under an atmosphere of dry argon using standard Schlenk techniques and vacuum-line manipulations unless stated otherwise. All solvents were distilled prior to use and were purified/dried according to standard procedures [28]. NMR spectra were recorded at room temperature with a Bruker Avance III 500 Ultra Shield Spectrometer (^1^H at 500.300 MHz and ^13^C{^1^H} at 125.813 MHz) in the Fourier transform mode. Chemical shifts are reported in δ (ppm) versus SiMe_4_ with the solvent as the reference signal ([D_6_]-DMSO: ^1^H NMR, δ = 2.54; and ^13^C{^1^H}NMR, δ = 40.45). FTIR spectra were recorded in the range of 400–4000 cm^−1^ on a Perkin-Elmer Spectrum 1000 FTIR spectrophotometer as KBr pellets. Elemental analysis for C, H and N were performed on a Thermo FlashAE 1112 series. The mononuclear Ni^II^-containing complexes [*n*-Bu_4_N]_2_[Ni(opboR_2_)] (R = Me, Et, *n-*Pr) were synthesized according to the literature [15]. Static magnetization measurements at *T* = 1.8 K and in magnetic fields µ_0_*H* up to 7 T were carried out with a 7 T VSM-SQUID magnetometer from Quantum Design. The temperature dependence of the static magnetization was measured in a temperature range *T* = 1.8–300 K and at µ_0_*H* = 1 T with this device. For these magnetic measurements, single crystals of the individual complexes were taken and gently heated (ca. 35 °C) overnight in vacuum to obtain materials free of packing solvents. Unfortunately, no inspection of the vacuum-dried crystals under the microscope was possible due to the hygroscopic nature of the materials, cf. below and Supporting Information File 1.

**Singe-crystal X-ray crystallographic studies.** Intensity data of **1’**, **2’** and **3’**, respectively, were collected on an Oxford Gemini S diffractometer with Cu Kα radiation. The structures were solved by direct methods and refined by full-matrix least-squares methods on *F*^2^ with the SHELX-2013 software [29]. All non-hydrogen atoms were refined anisotropically, and riding models were employed in the treatment of the hydrogen atom positions. Crystallographic data have been deposited at the Cambridge Crystallographic Data Center under the CCDC numbers 923899 (**1’**), 923898 (**2’**) and 923900 (**3’**). In case of **1’** one CH_2_Cl_2_ packing solvent molecule has been refined to an occupation factor of 0.75 (Cl7, Cl8, C61) and another CH_2_Cl_2_ packing solvent molecule (Cl5, Cl6, C64) has been refined disordered on two position with occupation factors of 0.75/0.25. In case of **2’** the two ClO_4_^–^ counter ions were both refined disordered on two position with occupation factors of 0.61/0.39 (Cl1, O5–O8) and 0.50/0.50 (Cl2, O9–O12), respectively. Crystals of **2’** were all twinned. The selected one was composed of two nearly equally populated domains covering ca. 98% of all measured reflections, which were simultaneously integrated to generate a hklf 5 file with the diffractometer software [30]. In the case of **3’**, the CH_2_Cl_2_ packing solvent molecule (Cl1, Cl2, C18) has been refined disordered on two position with occupation factors of 0.67/0.33.

**Synthesis of [NiCu_2_(opboR_2_)(pmdta)_2_][X]_2_**, **R = Me, X = NO_3_ (1); R = Et, X = ClO_4_** **(2), R = *n-*Pr, X = NO_3_ (3)**. To a solution of [*n-*Bu_4_N]_2_[Ni(opboR_2_)] (R = Me, *^n^*Pr) or [*n-*Bu_4_N]_2_[Ni(opboEt_2_)] (0.0006 mol) in MeCN (50 mL) a solution of [Cu(pmdta)(NO_3_)_2_] (0.0012 mol) in MeCN (25 mL) or [Cu(pmdta)(ClO_4_)_2_] (0.0012 mol) in MeCN (25 mL) was added, respectively. After stirring for 1 h, the resulting reaction mixture was concentrated to approximately 5 mL and Et_2_O (100 mL) was added to give a green precipitate. The overlaying solvent mixture was removed via a Teflon tube and MeCN (5 mL) was added to dissolve the residue. A mixture of THF/Et_2_O 4:1 (100 mL) was added to precipitate a green powder, which was washed twice with the same solvents mixture (50 mL). After removal of the supernatant, the remaining solid was dried in vacuum. Crystals suitable for X-ray crystallographic studies were grown by slow diffusion of Et_2_O vapour in CH_2_Cl_2_ solutions of **1** and **3** and in a MeCN solution of **2**. Supporting Information File 1 gives the IR spectra of **1**–**3**, respectively.

**1.** Yield: 0.35 g (63%); anal. calcd for C_30_H_56_Cu_2_N_12_NiO_10_ (930.63 g·mol^–1^): C, 38.72; H, 6.07; N, 18.06; found: C, 38.22; H, 5.85; N, 17.92%; IR: ν = 2958 (m), 2946 (m) (CH); 1630 (s), 1602 (m) (CO); (1383) (s) (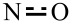).

**2.** Yield: 0.44 g (77%); anal. calcd for C_32_H_60_Cl_2_Cu_2_N_10_NiO_12_ (1033.57 g·mol^–1^): C, 37.19; H, 5.85; N, 13.55; found: C, 37.22; H 5.74; N, 13.28%; IR: ν = 2983 (m), 2960 (m) (CH); 1653 (m), 1614 (m) (CO); (1061) (s) (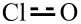).

**3.** Yield: 0.43 g (74%); anal. calcd for C_34_H_64_Cu_2_N_12_NiO_10_ (986.73 g·mol^–1^): C, 41.39; H, 6.54; N, 17.03; found: C, 41.11; H, 6.39; N, 16.89%; IR: ν = 2977 (m), 2951 (m) (CH); 1647 (s), 1614 (m) (CO); (1389) (s) (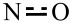).

## Supporting Information

File 1 IR spectra of **1**–**3**.
