# Supplementary material for: Probing the magnetic superexchange couplings between terminal CuII ions in heterotrinuclear bis(oxamidato) type complexes
Source: Beilstein J Nanotechnol. 2017 Apr 6;8:789–800. doi: 10.3762/bjnano.8.82 (PMC5389195; doi:10.3762/bjnano.8.82)
Supplement: File 1 — IR spectra of 1–3. [file Beilstein_J_Nanotechnol-08-789-s001.pdf]

Supporting Information

for

Probing the magnetic superexchange couplings between terminal Cu^{II} ions in heterotrinuclear bis(oxamidato) type complexes

Mohammad A. Abdulmalic[1], Saddam Weheabby[1], Francois E. Meva[2], Azar Aliabadi[3,4], Vladislav Kataev[3], Bernd Büchner[3,5], Frederik Schleife[6], Berthold Kersting[6] and Tobias Rüffer*[1,§]

Address: [1]Department of Inorganic Chemistry, Faculty of Natural Sciences, Chemnitz University of Technology, Strasse der Nationen 62, D-09111 Chemnitz, Germany, [2]Department of Pharmaceutical Sciences, Faculty of Medicine and Pharmaceutical Sciences, University of Douala, BP 2701, Cameroon, [3]Leibniz Institute for Solid State and Materials Research IFW Dresden, D-01171 Dresden, Germany, [4]Institut für Nanospektroskopie (EM-ISPEK), Helmholtz-Zentrum Berlin für Materialien und Energie, Kekuléstr. 5, D-12489 Berlin, Germany, [5]Institute for Solid State Physics, Technical University Dresden, D-01062 Dresden, Germany and [6]Department of Inorganic Chemistry, Faculty of Chemistry and Mineralogy, University of Leipzig, Johannisallee 29, D-04103 Leipzig, Germany

Email: Tobias Rüffer - tobias.rueffer@chemie.tu-chemnitz.de

* Corresponding author

§ Phone: +49 (0)371-531-31836

IR spectra of 1–3

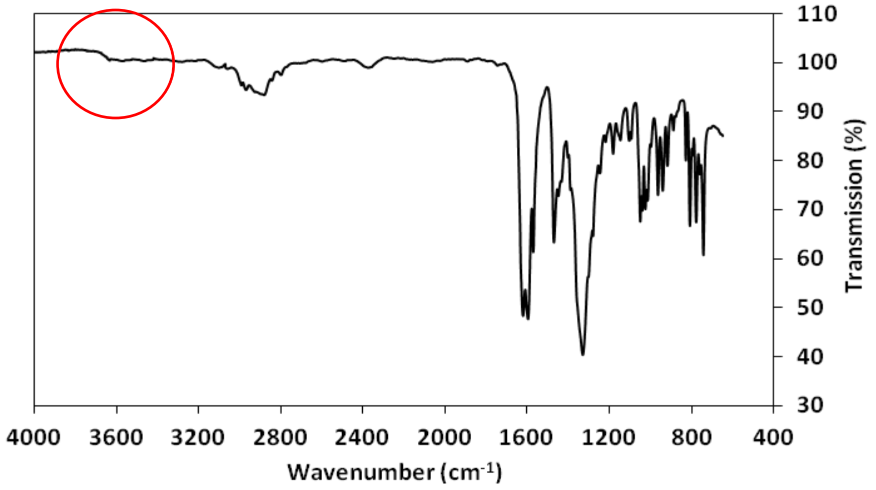

Figure S1: IR spectrum of **1**.

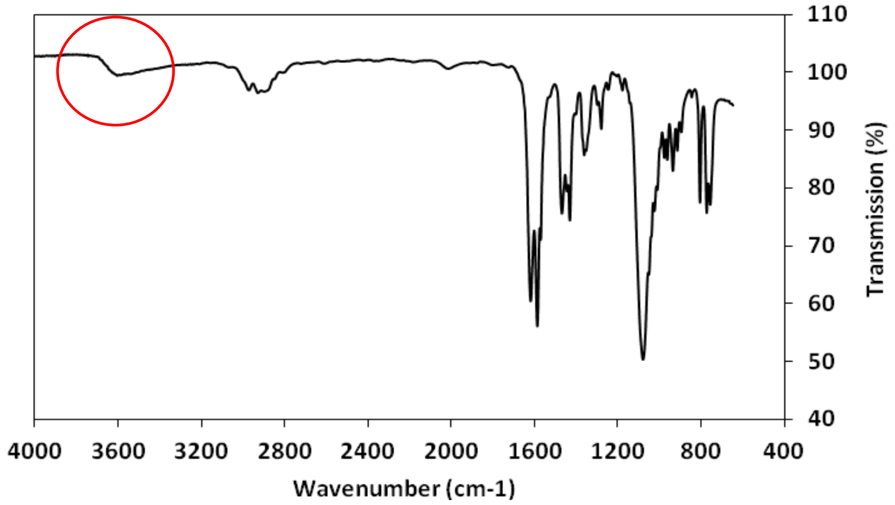

Figure S2: IR spectrum of **2**.

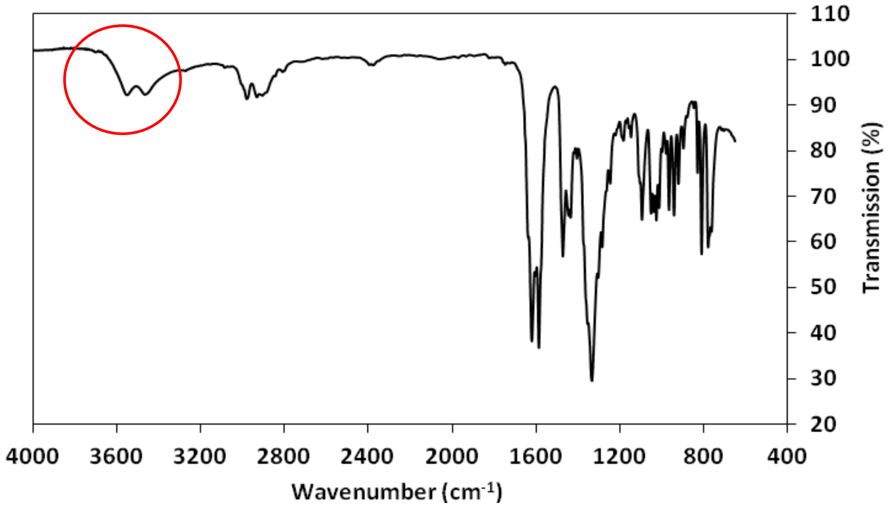

Figure S3: IR spectrum of **3**.

Note: Due to the aerobic sample handling for obtaining KBr tablets all three samples caught water (indicated by circles) out of air moisture as they are (extremely) hygroscopic, especially complex 3.

Table S1: Experimental and calculated $\chi_M T$ [cm^3·mol^{-1}·K] and $1/\chi_M$[mol/cm^3] values of **2**.

***T* / K**	**$\chi_M T$ (exp)**	**$\chi_M T$ (fit)**	**$1/\chi_M$ (exp)**	**$1/\chi_M$ (fit)**
2.00068	0.71488	0.71566	2.79862	2.79557
3.09532	0.74842	0.74562	4.13581	4.15134
3.98311	0.76130	0.75763	5.23198	5.25733
5.53291	0.76901	0.76949	7.19485	7.19036
6.00596	0.76553	0.77195	7.84549	7.78024
7.00808	0.77345	0.77615	9.06081	9.02929
8.00156	0.77879	0.77939	10.27435	10.26644
9.00345	0.78205	0.78205	11.51263	11.51263
10.00316	0.78417	0.78427	12.75637	12.75474
11.01117	0.78630	0.78619	14.00378	14.00574
11.99986	0.78700	0.78786	15.24760	15.23095
13.00332	0.78963	0.78936	16.46761	16.47324
14.00689	0.79174	0.79074	17.69127	17.71365
15.00135	0.79296	0.79198	18.91817	18.94158
16.00161	0.79379	0.79315	20.15849	20.17476
17.00127	0.79480	0.79424	21.39063	21.40571
18.00134	0.79555	0.79527	22.62754	22.63551
19.00144	0.79637	0.79625	23.86007	23.86366
19.99654	0.79710	0.79718	25.08661	25.08410
30.00246	0.80461	0.80511	37.28820	37.26504
39.99930	0.81109	0.81180	49.31549	49.27236
50.03473	0.81729	0.81802	61.22029	61.16566
60.09363	0.82323	0.82401	72.99738	72.92828
70.12439	0.82937	0.82985	84.55139	84.50249
80.16077	0.83554	0.83560	95.93888	95.93199
90.19693	0.84151	0.84129	107.18462	107.21265
100.2365	0.84713	0.84694	118.32481	118.35136
110.2696	0.85288	0.85256	129.29087	129.33940
120.2901	0.85831	0.85815	140.14762	140.17375
130.33749	0.86450	0.86374	150.76633	150.89899
140.36819	0.87033	0.86931	161.28157	161.47081
150.39101	0.87562	0.87486	171.75374	171.90294
160.42270	0.88102	0.88040	182.08747	182.21570
170.47180	0.88683	0.88595	192.22602	192.41695
180.50751	0.89265	0.89149	202.21533	202.47845
190.55800	0.89861	0.89703	212.05862	212.43214
200.56650	0.90369	0.90254	221.94171	222.22450
210.54221	0.90882	0.90803	231.66547	231.86702
220.51990	0.91393	0.91352	241.28752	241.39581
230.64191	0.91979	0.91909	250.75497	250.94595
240.45390	0.92414	0.92448	260.19207	260.09638
250.44949	0.92975	0.92997	269.37294	269.30921
260.44421	0.93522	0.93546	278.48443	278.41298
270.44989	0.94028	0.94095	287.62697	287.42217
280.43549	0.94559	0.94643	296.57197	296.30875
290.43491	0.95112	0.95192	305.36095	305.10433
300.43649	0.95644	0.95741	314.11954	313.80129
310.28259	0.96171	0.96281	322.63634	322.26773
320.44299	0.96771	0.96838	331.13535	330.90625
330.43301	0.97384	0.97386	339.30934	339.30237
